# Establishing the minimal clinically important difference of the 6-min walk test in lung transplant recipients undergoing pulmonary rehabilitation: a prospective analysis

**DOI:** 10.3389/fmed.2026.1740439

**Published:** 2026-01-21

**Authors:** Peijian Wang, Beiyao Gao, Jing Sun, Yang Yu, Lijun Ge, Shan Jiang, Siyuan Wang, Wenhui Chen

**Affiliations:** 1 Department of Rehabilitation Medicine, China-Japan Friendship Hospital, Beijing, China; 2 National Center for Respiratory Medicine, Beijing, China; 3 State Key Laboratory of Respiratory Health and Multimorbidity, Beijing, China; 4 National Clinical Research Center for Respiratory Diseases, Beijing, China; 5 Institute of Respiratory Medicine, Chinese Academy of Medical Sciences, Beijing, China; 6 Department of Lung Transplantation, Center of Respiratory Medicine, China-Japan Friendship Hospital, Beijing, China; 7 Department of Laboratory Medicine, China-Japan Friendship Hospital, Beijing, China

**Keywords:** exercise capacity, lung transplant recipients, minimal clinically important difference, pulmonary rehabilitation, 6-min walk test

## Abstract

**Purpose:**

This study aimed to evaluate the responsiveness of the 6-min walk test (6MWT) to pulmonary rehabilitation (PR) in lung transplant recipients (LTRs) and to establish the minimum clinically important difference (MCID) for the 6MWT in this population.

**Materials and methods:**

Eighty-one LTRs from a single-center randomized controlled trial were included. The MCID was calculated using anchor-based (handgrip strength) and distribution-based methods. Handgrip strength (left, right, and average) and 6MWT distance were assessed before and after an 8-week PR program.

**Results:**

All 81 participants completed PR. The 6MWT distance improved significantly, with a mean change of 73.37 m (Cohen’s *d* = 1.10). Handgrip strength increased by 7.24–8.21 kg (Cohen’s *d* = 0.80–0.91; all *p* < 0.001). Changes in handgrip strength were strongly correlated with changes in 6MWT distance (*r* = 0.63–0.69; *p* < 0.001). The MCID was estimated at 52.65–58.5 m (anchor-based), 57.28–59.40 m (distribution-based), and 56.83 m (combined).

**Conclusion:**

The 6MWT is responsive to PR in LTRs. A MCID of 56.83 m provides a clinically meaningful benchmark for interpreting exercise capacity changes and can guide sample size estimation in future trials.

## Introduction

Lung transplantation (LTx) serves as the definitive therapeutic intervention for patients with end-stage lung diseases. Advances in surgical techniques, perioperative management, and immunosuppressive protocols have significantly improved postoperative survival rates, with a median survival of 4–6 years (1). However, functional independent recovery remains a critical challenge in clinical practice. Following lung transplantation, there is a marked improvement in pulmonary function. However, lung transplantation recipients (LTRs) still experience physical impairments, such as limited exercise capacity (40–60% of predicted normal values), early-onset of metabolic acidosis and skeletal muscle weakness, all of which persist for years after transplant surgery (2). Numerous studies reported that exercise capacity is not only a core indicator of functional recovery but also an important predictor of mortality and has been established as a predictor of post-transplant survival (3–5).

Pulmonary rehabilitation (PR), a multidisciplinary intervention, has demonstrated efficacy in improving exercise tolerance, quality of life, and mental health in chronic respiratory disease management (6). A meta-analysis of 21 studies (1,579 participants) further revealed that PR significantly enhances 6-min walk test (6MWD) in LTRs. Nevertheless, the study also emphasized the necessity to establish the minimum clinically important difference (MCID) for the 6-min walk test (6MWT) in this population to precisely evaluate the clinical efficacy of PR (7). The MCID serves to evaluate the effectiveness of particular treatments, helps in comprehending the clinical relevance of statistically significant outcomes, and is useful for determining sample size (8, 9). Although one study has reported the MCID for the 6MWT in LTRs to be 70 m using the distribution—based method, further validation with the anchor—based approach is still needed. To address this need, we used handgrip strength as an objective anchor for MCID estimation, given its ability to reflect disease pathophysiology and provide clinically meaningful insights (10, 11). Handgrip strength, a validated indicator of overall muscle function that strongly correlates with 6MWD in LTRs (12, 13), was therefore selected as a biologically plausible anchor for interpreting clinically meaningful changes in walking capacity.

Based on this rationale, this study aimed to evaluate the responsiveness of the 6MWT to PR in LTRs and to develop a multidimensional MCID model integrating anchor-based and distribution-based approaches for a comprehensive assessment of clinical significance.

## Methods

This study is part of a single center randomized controlled trial (ChiCTR2200063538), which was approved by Clinical Research Ethics Committee of China-Japan Friendship Hospital (No. 2022-KY-148) and the date of first registration was 10/Sep/2022.

### Participants

Participants were recruited between October 2022 and May 2025 at the China-Japan Friendship Hospital. Inclusion criteria were as follows: (1) aged 18 years or older; (2) undergone unilateral or bilateral lung transplantation; (3) able to stand unsupported for at least 5 min; (4) able to walk 50 m or more (assistive devices permitted); (5) provided written informed consent. The exclusion criteria referred to post-transplant conditions and were established to ensure patient safety and adherence during PR. Patients were excluded if they met any of the following: (1) presence of severe comorbidities affecting major organ systems (cardiac, cerebral, hepatic, or renal dysfunction); (2) psychiatric, cognitive, or musculoskeletal disorders that would impair adherence to pulmonary rehabilitation protocols; (3) clinically unstable conditions deemed rehabilitation-contraindicated by physicians; (4) inability to provide informed consent; (5) noncompliance with prescribed PR protocols.

#### PR programs

All LTRs received standardized postoperative care, which included infection control, individualized immunosuppressive therapy, and vital organ support. During the same period, participants underwent a 12-week structured PR program (3–5 days per week) consisting of three integrated phases:

1. Endurance Training: Each endurance training session (treadmill walking) began with a 5-min warm-up period, including short active stretching exercises for the biceps, neck, shoulder, trunk, calves, hamstrings, and quadriceps. The initial treadmill speed was set at approximately 70% of the average speed achieved during the baseline 6MWT. For participants unable to tolerate continuous endurance exercises, interval exercises were used instead. The speed was adjusted weekly based on the patient’s tolerance, with the goal of progressively increasing the intensity while ensuring patient safety and comfort. Each session lasted 15–30 min.
2. Inspiratory Muscle Training: The inspiratory muscle training began with the resistance set at 30% of the maximal inspiratory mouth pressure (MIP). The resistance was gradually increased by 5% each week, aiming to strengthen the inspiratory muscles and enhance respiratory function. The training sessions were closely monitored to ensure proper technique and avoid any potential adverse effects
3. Resistance Training: Participants performed upper and lower limbs resistance training, consisting of 3 sets of 15 repetitions at moderate intensity (3–5 on Borg CR10 scale), with 1 min of rest between sets. The exercise load was progressed based on each patient’s individual tolerance, with careful attention to their form and fatigue levels. The goal was to build muscle strength and endurance while minimizing the risk of injury.

### Measurements

All baseline assessments were performed prior to the initiation of the PR program. Demographic data, resting oxygen saturation, transplant type (single/double LTx), lung function, dyspnea severity (via the Borg scale), interval between LTx and PR, duration in intensive care unit. Lung function, specifically forced expiratory volume in the first second (FEV1) and forced vital capacity (FVC), was measured via spirometry. The 6MWT and handgrip assessments were conducted pre and post the PR protocols. The assessment order was randomized, with a minimum 48-h interval.

#### 6MWT

We conducted the 6MWT in accordance with the guidelines established by the American Thoracic Society (14). Participants were asked to wear loose, comfortable clothing and comfortable footwear. They also should avoid testing on an empty stomach or after eating a full meal and should not engage in vigorous exercise within 2 h prior to the test. Before the test begins, researchers provided participants with a detailed explanation of the testing procedure and may demonstrate it if necessary. Baseline measurements, including blood pressure, heart rate, oxygen saturation, and Borg score, would be taken prior to the start of the test. During the test, participants were instructed to walk at the fastest possible pace along a 30-m indoor straight track to cover the maximum distance possible within 6 min. Test administrators will employ standardized verbal cues, such as “Keep going” or “You are doing well,” to encourage participants throughout the test. If a participant needed to rest due to fatigue, they may pause briefly but must resume walking as soon as they are able to continue.

#### Handgrip assessment

Handgrip strength (left, right, and average) were assessed using a handheld dynamometer (Jamar; Lafayette Instrument Company, Lafayette, IN). Patients were asked to sit in an upright position without back support, with both feet on the floor, elbows flexed at 90 degrees, and wrists in a neutral position. After familiarizing themselves with the procedure, they performed three maximum grip strength tests, alternating between the right and left hand (with a 20-s rest between each test). If the best two tests varied by more than 10%, an additional test was conducted to achieve a consistent maximum (15).

#### Statistics

The results of this study were presented as mean and standard deviation (SD), median and interquartile range, or proportions, depending on the appropriateness. Normality testing was performed on continuous variables. Chi-square tests, paired-samples *t*-tests, McNemar tests, or Mann–Whitney *U* tests were used to evaluate the differences following pulmonary rehabilitation, as per their suitability.

**Figure 1:**
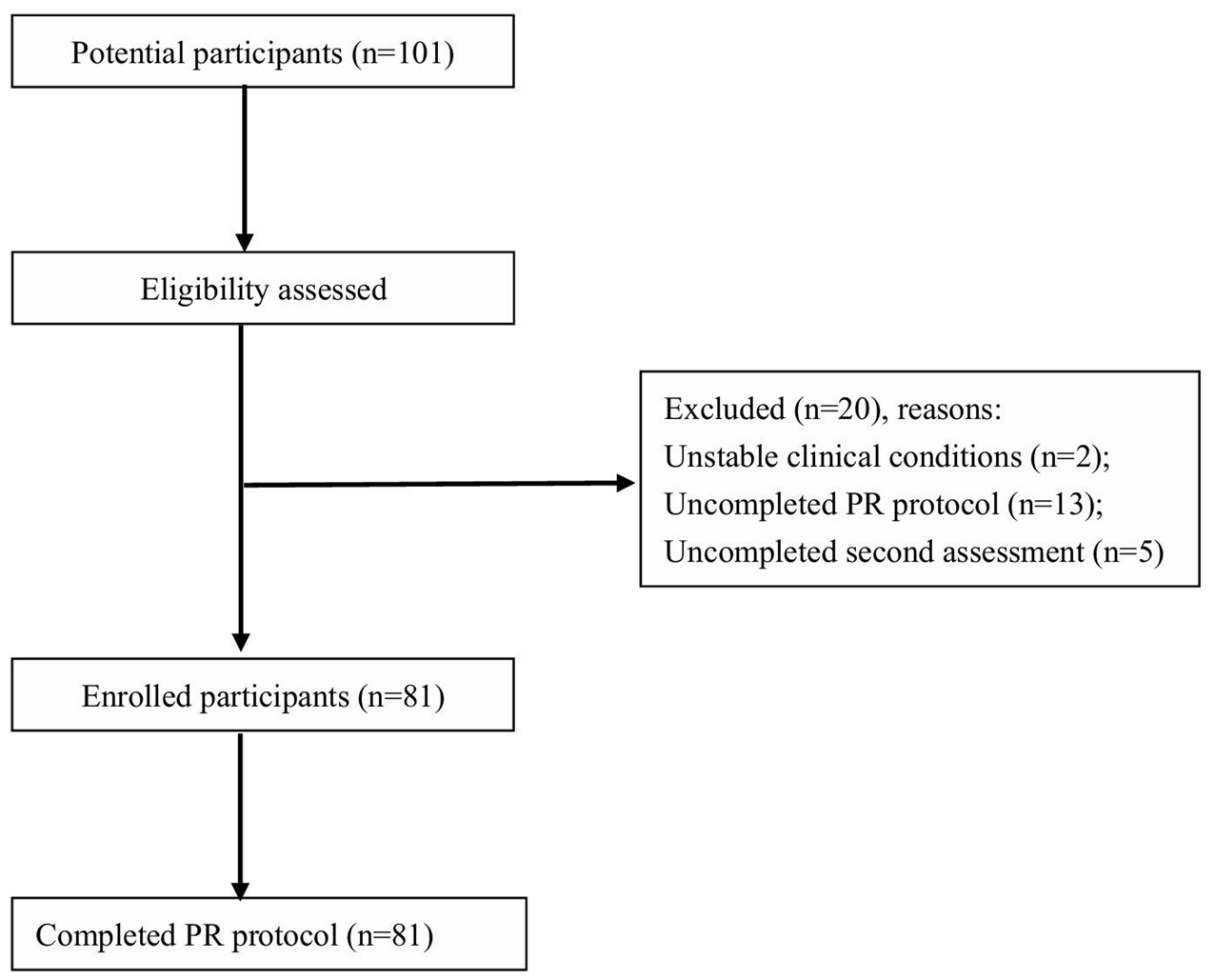
Participant screening and enrollment. PR, Pulmonary Rehabilitation.

To estimate the MCID, both anchor-based and distribution-based methods were used. As anchors, we referred to previously reported MCID values for handgrip strength (left hand: 4.03 kg; right hand: 4.95 kg; average: 3.93 kg) (12). Before using them as objective anchors, we calculated Pearson correlation coefficients (if data were normally distributed) to decide whether handgrip could be seen as anchors (*r* ≥ 0.3). For the anchor-based method, a linear regression analysis was performed, with the change in 6MWD as the dependent variable and the handgrip anchors (left, right, and average) as independent variables. In current study, we chosen two distribution—based approaches. First, we used the effect size (ES) as an intermediary metric. A moderate (0.5) Cohen’s ES was used to calculate the MCID (16). The second approach involves the standard error of measurement (SEM) and the reliability coefficient of the measurement instrument was derived from the previous study (17). To estimate the MCID of the current study, the mean of all MCID estimates was calculated. A *p* value < 0.05 was considered statistically significant. All statistics were done using SPSS V.25.0 (Figure 1).

## Result

A total of 101 LTRs were enrolled and 81participants completed the intervention in this study. 20 subjects withdrew from the study, with reasons including clinically unstable conditions (*n* = 2), failure to complete the PR program due to transportation issues (*n* = 13), and non-completion of the secondary assessment (6MWT or handgrip) (*n* = 5). Table 1 presented the baseline demographics of these patients. The age of the study population was 57.23 (12.77) years, with 71.6% being male and a mean body mass index (BMI) of 21.65 (3.26) kg/m^2^. The majority (86.4%) underwent double lung transplantation (DLT). The primary diseases leading to lung transplantation were interstitial lung disease (ILD) (40.74%), idiopathic pulmonary fibrosis (IPF) (22.22%), and COPD (19.75%). Moreover, 69.14% of LTRs had exercise-induced desaturation (EID) during 6MWT measured before the initiation of the pulmonary rehabilitation program. And EID was defined as SpO_2_min < 90% or a decline of ≥4% during 6MWT (18). At baseline pulmonary function assessment, the mean FEV1 and FVC percentage of predicted values were 55.13 and 57.79%, respectively.

**Table 1:** Baseline demographic and clinical characteristics of the study participants.

General characteristics	
Number (*n*)	81
Gender, male, *n* (%)	58 (71.60)
Age, y	57.23 ± 12.77
BMI (kg/m^2^)	21.65 ± 3.26
DLT	70 (86.42%)
Disease causing LTx, *n* (%)	
ILD	33 (40.74)
IPF	18 (22.22)
COPD	16 (19.75)
BOS	4 (4.94)
PF	3 (3.70)
PH	3 (3.70)
Others	4 (4.94)
EID	56 (69.14)
LTx to baseline assessment (d)	159.53 ± 155.15
Lung function at baseline	
FEV_1_ (L)	1.69 ± 0.56
FEV1 (% predicted)	55.13 ± 20.62
FVC (L)	2.11 ± 0.62
FVC (% predicted)	57.79 ± 18.87
FEV1/FVC	0.79 ± 0.13

Values are presented as mean ± standard deviation, median (interquartile range), or number (percentage), as appropriate. LTRs, lung transplantation recipients; LTx, lung transplantation; BMI, body mass index; DLT, double lung transplantation; ILD, interstitial lung disease IPF, idiopathic pulmonary fibrosis; COPD, chronic obstructive pulmonary disease; PF, pulmonary fibrosis; PH, pulmonary hypertension; EID, exercise-induced desaturation; BOS, bronchiolitis obliterans syndrome; FEV_1_, forced expiratory volume in the first second; FVC, forced vital capacity.

Table 2 illustrated the mean changes in 6-min walk distance (6MWD) and handgrip strength before and after the PR program. The data demonstrates a clear and significant improvement in both exercise capacity and muscle strength, highlighting the effectiveness of the rehabilitation intervention (*d* = 0.80–1.10).

**Table 2:** Changes between pre and post intervention.

Outcome	Pre-rehabilitation	Post-rehabilitation	Mean change	Cohen *d*	ES	*P* value
Handgrip, kg						
Left	16.66 ± 6.47	23.90 ± 9.11	7.24 ± 9.09	0.80	1.12	<0.001
Right	19.10 ± 7.00	27.31 ± 10.46	8.21 ± 9.07	0.91	1.17	<0.001
Average	17.88 ± 6.46	25.61 ± 9.30	7.73 ± 8.44	0.92	1.20	<0.001
6MWT, m	351.69 ± 114.55	425.06 ± 98.81	73.37 ± 66.40	1.10	0.64	<0.001

Values are presented as mean ± standard deviation. 6MWT, 6-min walk test; ES, effect size.

**Figure 2:**
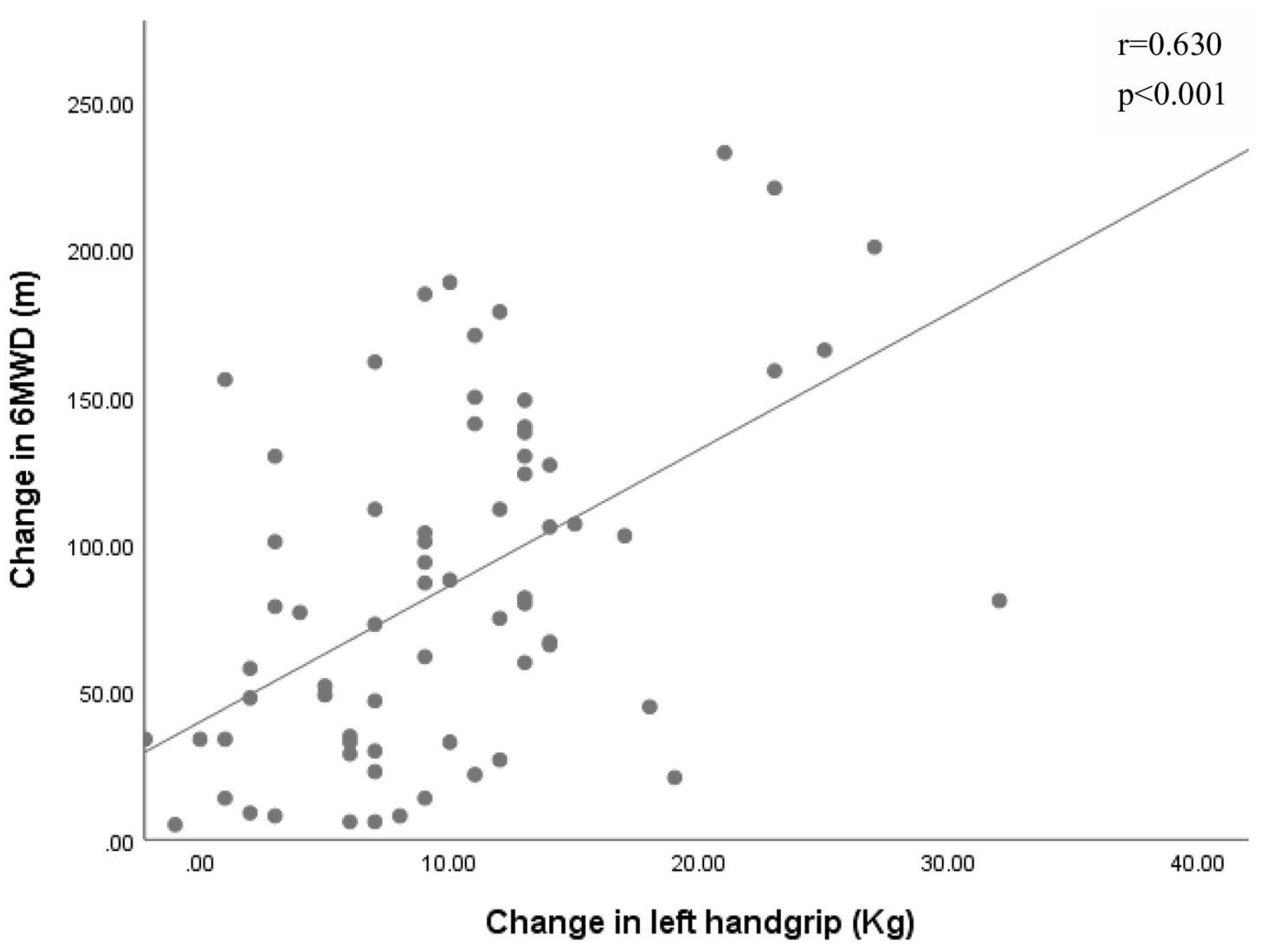
Association between the changes in left handgrip and the change in 6-min walk distance (6MWD).

**Figure 3:**
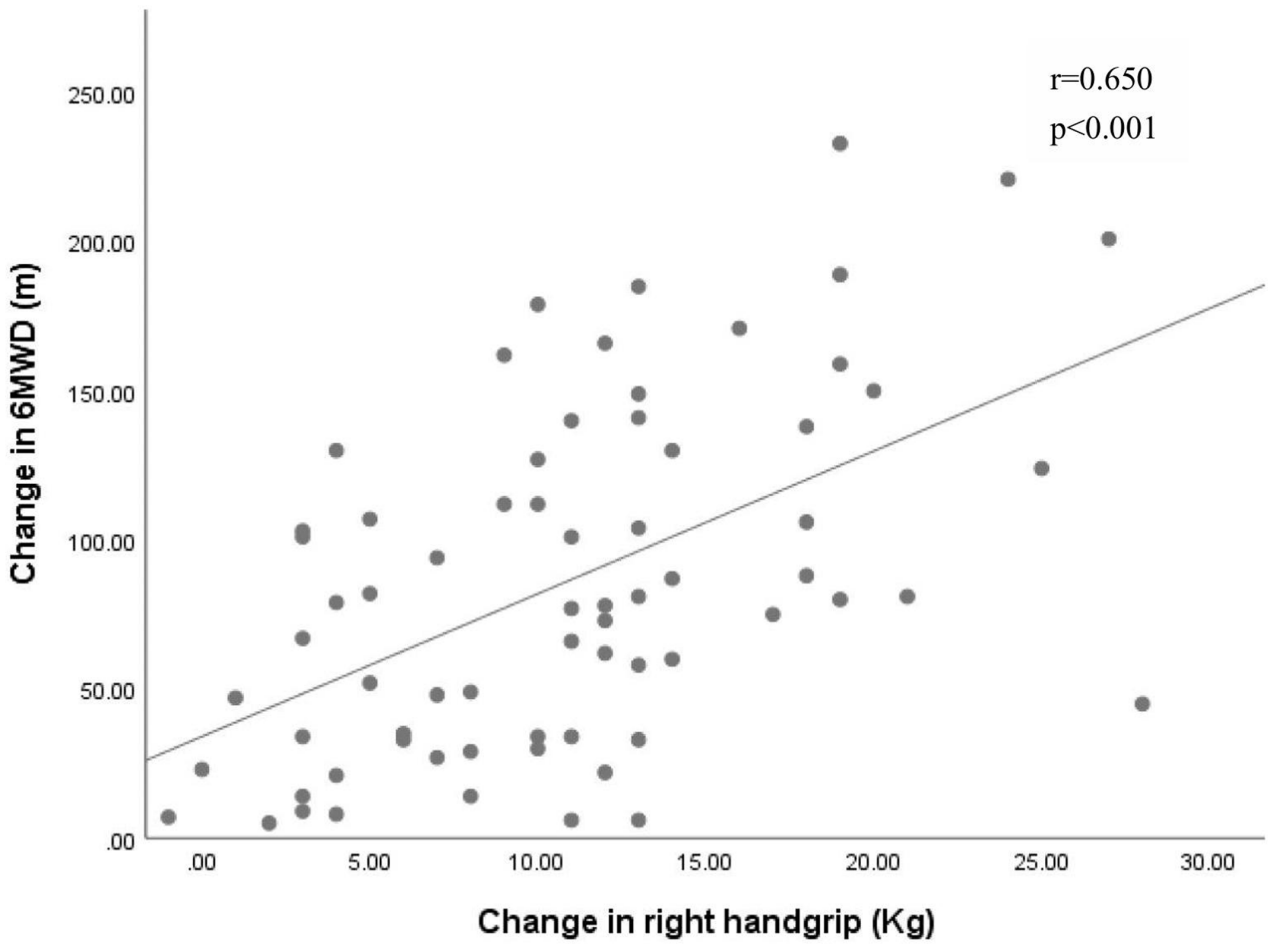
Association between the changes in right handgrip and the change in 6MWD. 6MWD, 6-min walk distance.

**Figure 4:**
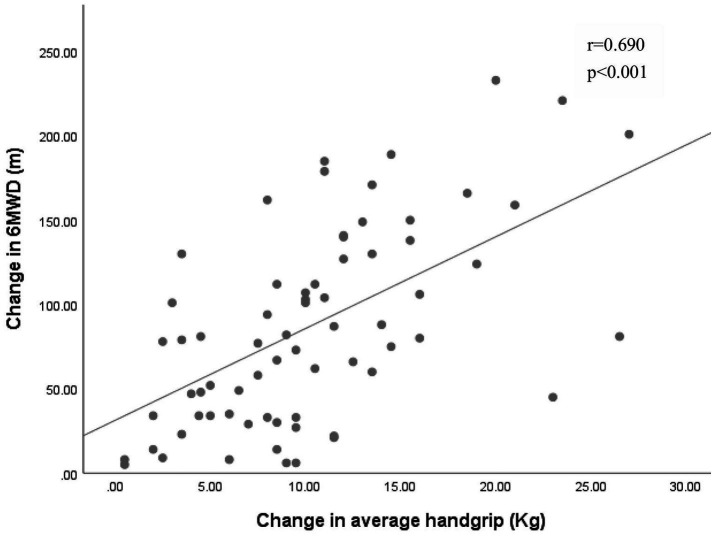
Association between the changes in average handgrip and the change in 6MWD. 6MWD, 6-min walk distance.

Pearson correlation analysis revealed significant associations between changes in grip strength (left: *r* = 0.63, right: *r* = 0.65, average grip: *r* = 0.69) and improvements in the 6MWT (*p* < 0.001 for all) (Figures 2–4).

### MCID estimation for 6MWT

The outcomes of the calculations for the anchor-based, distribution-based and combined methods were shown in Table 2. We calculated a 6MWT MCIDs of each anchor: left handgrip 58.50 m, right handgrip 56.30 m and average handgrip 52.65 m. With the 0.5*SD_pre_ distribution-based method, 57.28 m was observed as the MCID. The SEM distribution-based method estimated the 6MWT MCID at 59.40 m. The combined MCID (average of the three anchor-based MCIDs and two distribution-based MCID) was 56.83 m.

## Discussion

To the best of our knowledge, we established for the first time a 6MWT MCID specifically using combination methods in LTRs. The current study provided valuable insights into the responsiveness of the 6MWT to PR in LTRs and offered a robust estimation of the MCID for the 6MWT in this specific patient population. The average MCID of 56.83 m identified in this study serves as a clinically meaningful threshold for interpreting improvements in exercise capacity following PR interventions.

### Responsiveness of the 6MWT to PR in LTRs

Our study confirmed that the 6MWT is highly responsive to PR in LTRs, with a large effect size (Cohen’s *d* = 1.10). Notably, despite a longer PR intervention duration (12 weeks vs. 8 weeks in a prior study), the mean improvement in 6MWT distance (73.37 m) was smaller than the 174.2 m reported previously (17). Two key factors may explain this discrepancy: first, demographic and clinical differences between cohorts, including older mean age (57.23 years vs. 38.4 years) and a longer interval from LTx to postoperative PR initiation (159.53 days vs. 31.4 days), which have been shown to modulate PR outcomes in LTRs (7); second, a high proportion of our participants (69.14%) had EID at baseline—a condition known to impact functional outcomes in chronic respiratory diseases (19, 20), though its specific effect on PR responsiveness in LTRs warrants further investigation.

Importantly, the interval between lung transplantation and baseline assessment varied across participants, indicating enrollment at different postoperative stages. This reflects real-world clinical practice, in which PR is initiated once clinical stability is achieved rather than at a fixed postoperative time point. Although recovery trajectories and responsiveness to rehabilitation may differ by postoperative stage, the present analysis focused on within-subject pre–post changes in 6MWT distance. This self-controlled design minimizes confounding from baseline functional heterogeneity and transplant timing, thereby facilitating the assessment of PR-related improvement across a heterogeneous post-transplant population.

### MCID estimate

This study estimated the 6MWT for the MCID to be 56.83 m in postoperative LTRs, which is smaller than the 79 m reported in a prior study using a single distribution-based method (17). Three interrelated factors contribute to this discrepancy: first, MCID estimates are known to be influenced by disease severity and clinical complexity (21, 22), and our cohort included older LTRs (mean age 57.23 years vs. 38.4 years) with higher baseline complexity (69.14% with EID), which likely reduced the magnitude of PR-induced 6MWT improvement and consequently yielded a lower MCID; second, unlike the prior study that relied solely on a distribution-based approach, we adopted a combined anchor-based (handgrip strength) and distribution-based method, which addresses the limitations of single-method estimates and enhances result robustness (16, 23); third, the integration of clinical relevance and statistical rigor avoids the potential overestimation associated with standalone distribution-based calculations (12, 13).

The 56.83 m MCID holds important clinical and research implications: first, it exceeds the MCID for exercise capacity in chronic respiratory diseases (e.g., 26–30 m in COPD) (21), highlighting that LTRs require a greater improvement in 6MWT distance to achieve clinically meaningful benefits from PR—reflecting the unique physiological challenges of post-transplant recovery; second, it provides a practical clinical benchmark: a 6MWT improvement exceeds 56.83 m post-PR can be defined as a “clinically effective response,” enabling clinicians to identify non-responders and adjust rehabilitation protocols in a timely manner; third, it supports rigorous trial design by offering a validated threshold for sample size calculation, reducing the risk of underpowered studies in LTR rehabilitation research.

### Methodological considerations

Several methodological considerations should be acknowledged. First, handgrip was selected as the anchor for MCID estimation, given its close association with skeletal muscle dysfunction in LTRs and its strong correlation with the 6MWD. Although this objective measure strengthens the pathophysiological relevance of the estimates, it does not fully capture patients’ perceived improvement—a core aspect of the MCID concept (10). Considering the heterogeneity in anchor selection and the uncertainty in defining a minimal important difference, future studies should employ multiple anchors (both subjective and objective) to reduce estimation error and enhance robustness. Secondly, we conducted the 6MWT only before and after intervention, and the intraclass correlation coefficient (ICC) for measuring the MCID based on the SEM was derived from a previous study (17). While we did not anticipate that this approach significantly affected the current results, the reliability of the findings would have been enhanced if the ICC had been obtained from the same sample as the standard deviation (24). Thirdly, there is a lack of a universally accepted standard for determining the MCID. Different calculation methods may yield varying results. A MCID that is estimated to be too low might lead to an overestimation of the effects of an intervention. Conversely, a MCID that is estimated to be too high may result in patients who actually benefit from an intervention being incorrectly judged as not responding to it in clinical practice (9). Although, many studies recommend using a combination of different methods to determine the MCID. There is currently no robust research on the appropriate weight allocation. We synthesized methodologies from existing studies and ultimately implemented a mean-weighted approach to calculate the MCID (16, 23). Finally, while anchor-based methods (e.g., linear regression) prioritize clinical interpretability of MCID, their validity remains limited by anchor selection, analytical approaches (absolute/relative change), and population heterogeneity (e.g., disease severity, interventions) (25). Although our study demonstrated significant correlations between grip strength (anchor) and 6MWT improvements, future studies should integrate multiple validated anchors and reduce inter-subject variability to enhance MCID reliability.

## Conclusion

In conclusion, the 6MWT is a responsive and reliable measure of exercise capacity in LTRs undergoing PR. Besides, our new MCID estimates (56.83 m) could be applied for both interpreting improvements in 6MWT distance as well as sample size determination in future clinical trials focus on investigating LTRs.

## Data Availability

The raw data supporting the conclusions of this article will be made available by the authors, without undue reservation.
