# Correction: Establishing the minimal clinically important difference of the 6-min walk test in lung transplant recipients undergoing pulmonary rehabilitation: a prospective analysis

**DOI:** 10.3389/fmed.2026.1794258

**Published:** 2026-03-18

**Authors:** Peijian Wang, Beiyao Gao, Jing Sun, Yang Yu, Lijun Ge, Shan Jiang, Siyuan Wang, Wenhui Chen

**Affiliations:** 1 Department of Rehabilitation Medicine, China-Japan Friendship Hospital, Beijing, China; 2 National Center for Respiratory Medicine, Beijing, China; 3 State Key Laboratory of Respiratory Health and Multimorbidity, Beijing, China; 4 National Clinical Research Center for Respiratory Diseases, Beijing, China; 5 Institute of Respiratory Medicine, Chinese Academy of Medical Sciences, Beijing, China; 6 Department of Lung Transplantation, Center of Respiratory Medicine, China-Japan Friendship Hospital, Beijing, China; 7 Department of Laboratory Medicine, China-Japan Friendship Hospital, Beijing, China

**Keywords:** exercise capacity, lung transplant recipients, minimal clinically important difference, pulmonary rehabilitation, 6-min walk test

The order of authors in the author list of the published paper was erroneously given as: Peijian Wang^1^, Beiyao Gao^1^, Jing Sun^2,3,4,5,6^, Yang Yu^7^, Lijun Ge^1^, Wenhui Chen^2,3,4,5,6*^, Shan Jiang^1*^, Siyuan Wang^1*^

The correct author list reads: Peijian Wang^1^, Beiyao Gao^1^, Jing Sun^2,3,4,5,6^, Yang Yu^7^, Lijun Ge^1^, Shan Jiang^1*^, Siyuan Wang^1*^ and Wenhui Chen^2,3,4,5,6*^

The original version of this article has been updated.

